# Association of bacteria in pancreatic fistula fluid with complications after pancreatic surgery

**DOI:** 10.1002/bjs5.50272

**Published:** 2020-04-16

**Authors:** E. Demir, K. Abdelhai, I. E. Demir, C. Jäger, F. Scheufele, S. Schorn, K. Rothe, H. Friess, G. O. Ceyhan

**Affiliations:** ^1^ Department of Surgery Munich Germany; ^2^ Institute for Microbiology Immunology and Hygiene, Klinikum rechts der Isar, Technical University of Munich, School of Medicine Munich Germany; ^3^ Hepato‐Pancreato‐Biliary Unit, Department of General Surgery Acibadem Mehmet Ali Aydinlar University School of Medicine Istanbul Turkey

## Abstract

**Background:**

Pancreatic fistula (PF) is a common complication after pancreatic surgery. It is unclear how microbes in PF fluid affect outcomes and which microbes are present after pancreatoduodenectomy (PD) and distal pancreatectomy (DP). The aim of this study was to compare the microbiological spectrum of PF fluid after PD *versus* DP, and its association with postoperative complications.

**Methods:**

Bacterial strains and antibiotic resistance rates of bacterial swabs obtained from the PF fluid of patients who underwent DP or PD were analysed. Cultured bacteria were classified as Enterobacterales and as ‘other intestinal and non‐intestinal microorganisms’ based on whether they are typically part of the normal human intestinal flora.

**Results:**

A total of 847 patients had a pancreatic resection (PD 600; DP 247) between July 2007 and December 2016. Clinically relevant PF was detected in 131 patients (15·5 per cent). Bacterial swabs were obtained from 108 patients (DP 47; PD 61), of which 19 (17·6 per cent) were sterile. Enterobacterales were detected in 74 per cent of PF fluid swabs after PD, and in 34 per cent after DP. Infected, polymicrobial or multidrug‐resistant PF fluid was more common after PD (rates of 95, 50 and 48 per cent respectively) than after DP (66, 26 and 6 per cent respectively). Patients with higher grade complications (Clavien–Dindo grade IV–V) or grade C PF had more Enterobacterales and multidrug‐resistant Enterobacterales in the PF fluid after DP.

**Conclusion:**

Enterobacterales and multidrug‐resistant bacteria are detected frequently after PD and DP, and are associated with more severe complications and PF in patients undergoing DP.

## Graphical abstract

This study showed that, despite the absence of intestinal lumen opening, pancreatic fistulas after distal pancreatectomy frequently contain intestinal bacteria, which markedly worsen the clinical outcome. Understanding the pathogenetic mechanism behind this high prevalence of intestinal bacteria within distal pancreatectomy‐associated pancreatic fistulas may help to generate effective therapeutic regimens.

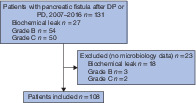

Enterobacteria associated with complications after distal pancreatectomy

## Abstract (es)

**Antecedentes:**

La fístula pancreática (*pancreatic fistula*, PF) es una complicación frecuente de la cirugía pancreática. No está claro cómo los microorganismos que se encuentran en el líquido de la PF (*pancreatic fistula fluid*, PFF) afectan los resultados y qué microbios están presentes después de la duodenopancreatectomía (*pancreaticoduodenectomy*, PD) y de la pancreatectomía distal (*distal pancreatectomy*, DP). El objetivo de este estudio fue comparar el espectro microbiológico del PFF después de PD *versus* DP y su asociación con las complicaciones postoperatorias.

**Métodos:**

Se analizaron las cepas bacterianas y las tasas de resistencia a los antibióticos de las muestras bacterianas obtenidas del PFF de pacientes de nuestra institución que se sometieron a DP o PD. Las bacterias identificadas en los cultivos se clasificaron en "enterobacterias" y "otros microorganismos intestinales y no intestinales" en función de si típicamente forman parte de la flora intestinal humana normal o no.

**Resultados:**

Un total de 847 pacientes se sometieron a resección pancreática (PD: 600, DP: 247) entre julio de 2007 y diciembre de 2016, y se detectó FP clínicamente relevante en 131 pacientes (15,5%). Se obtuvieron muestras bacterianas de 108 pacientes (DP n = 47, PD N = 61), de los cuales 19 (18%) eran estériles. Se detectaron enterobacterias en el 74% del PFF después de PD y en el 34% después de DP. El PFF infectado, con flora polimicrobiana o flora multirresistente fue más frecuente después de la PD (95,1%, 50%, 47,5%, respectivamente) que después de la DP (66,0%, 25,8%, 6,4%, respectivamente). Los pacientes con complicaciones de grado superior (Clavien‐Dindo 4/5) o PF grado C presentaron más enterobacterias y enterobacterias multirresistentes en el PFF después de DP.

**Conclusión:**

Las enterobacterias y las bacterias multirresistentes se detectaron con frecuencia después de la PD y la DP, y se asociaron a complicaciones más graves y PF en pacientes sometidos a DP.

## Introduction

Pancreatic fistula (PF) continues to be among the most enigmatic complications after pancreatic surgery. Despite recent developments in surgical technique, its prevalence is still high, reaching 30–40 per cent after distal pancreatectomy (DP)^1^. In comparison, the prevalence of PF after pancreatic head resection/pancreatoduodenectomy (PD) is somewhat lower, around 15 per cent^2^. Several technical modifications have been tried to reduce the high PF rates after DP, including stapling, combination of suturing with stapling, patch application, stapler reinforcements, and pancreatojejunal anastomosis^1^. To date, none of these techniques has proven to reduce the incidence of PF consistently.

The leading reasons for PFs being a clinical problem are that they either become infected or cause erosive bleeding. In DP, surgeons typically do not open the intestinal lumen, in contrast to pancreatic head resection, which requires the creation of a pancreatojejunal or pancreatogastric anastomosis. Thus, it is unclear why PF after a sterile operation such as DP becomes infected, especially when most indications for DP are ‘sterile’ diseases such as pancreatic tail tumours.

To determine the reason behind the infection of PF after DP, the types of bacteria that are commonly detected in the microbiological analysis of PF fluids after DP need to be analysed. Thus, the primary aim of this study was to compare the bacterial spectrum of PF fluid after DP and PD, and analyse its association with complications.

## Methods

Data for patients who had surgery at the Department of Surgery, Klinikum rechts der Isar, Technical University of Munich, Germany, between 1 July 2007 and 31 December 2016 were obtained from a departmental database. Pancreatic resections were performed by seven experienced surgeons. Pancreatic stump closure during DP was performed by either hand‐sewn sutures or a stapler device, or a combination of both, but not via anastomosis. PF grades were defined according to the definition of the International Study Group on Pancreatic Surgery^3^.

Microbiology reports were screened for every patient included in the database. The indication for obtaining a swab of the PF fluid in all patients was suspected infection in the drain fluid in conjunction with fever and/or increased blood leucocyte count and/or C‐reactive protein concentration. Microbiological swabs were taken either from the drain fluid flowing over the abdominal drain placed during surgery, or after surgery from abdominal drains placed interventionally (for instance, CT‐guided). All documented microorganisms from the microbiology reports were collected in a second database. Perioperative single‐shot antibiotic prophylaxis with ampicillin plus sulbactam was given to every patient. No patient in the DP cohort had preoperative endoscopic retrograde cholangiopancreatography (ERCP) or preoperative transgastric biopsy.

The following microorganisms were classified as intestinal, based on their known presence in the normal intestinal flora: Enterobacterales (*Escherichia coli*, *Klebsiella* species (spp.), *Proteus* spp., *Citrobacter* spp.), *Enterococcus* spp., *Candida* spp., *Bacteroides* spp. and *Prevotella* spp.

The following strains were classified as ‘not primarily intestinal’: *Staphylococcus aureus,* coagulase‐negative staphylococci including *Staphylococcus epidermidis, Streptococcus* spp., *Corynebacterium* spp. and *Haemophilus parainfluenzae. Streptococcus* spp. and *H. parainfluenzae* can be encountered in the gastrointestinal tract, but are typically associated with skin and mucosa^4^, ^5^.

The occurrence of bacterial strains was correlated with the presence and severity of further complications such as haemorrhage, intra‐abdominal abscess, wound infection and length of hospital stay, and subanalysed based on the Clavien–Dindo classification of surgical complications^6^. The presence of antibiotic resistance in the collected PF fluid was also registered, using European Centre for Disease Prevention and Control definitions for the classification of bacterial resistance for Enterobacterales^7^. The incidence of Enterobacterales and of multidrug‐resistant Enterobacterales (MDRE) was compared between DP and pancreatic head resection.

### Statistical analysis

Statistical analysis was performed using IBM SPSS® Statistics version 25 (IBM, Armonk, New York, USA). Continuous data are presented as median (range) or median (i.q.r.) values, and categorical data are given as numbers and percentages. Statistical significance was tested with the χ^2^ test or Fisher's exact test, or with the Mann–Whitney *U* test, as appropriate. To identify risk factors, odds ratios (ORs) were calculated in univariable and multivariable logistic regression models. A two‐sided 95 per cent confidence interval with a significance level (*P* value) of 0·050 was determined for all calculations.

## Results

A total of 847 patients underwent pancreatic resection (PD, 600; DP, 247) between 1 July 2007 and 31 December 2016 in the authors' institution. Clinically relevant PF was detected in 131 patients, giving an overall fistula rate of 15·5 per cent (PD: biochemical leak 9 (1·5 per cent), grade B 25 (4·2 per cent), grade C 37 (6·2 per cent); DP: biochemical leak 17 (6·9 per cent), grade B 25 (10·1 per cent), grade C 18 (7·3 per cent)). Data for stiffness of the pancreas and pancreatic duct diameter in patients with PF are shown in *Table* *S1* (supporting information). Drains placed during surgery were left *in situ* for a mean of 7·9 (median 6) days. Interventionally placed drains stayed *in situ* for a mean of 14·1 (median 11) days.

**Figure 1:**
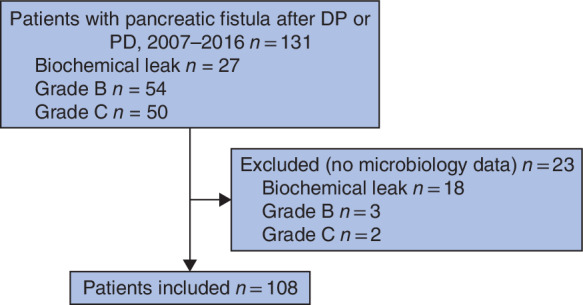
Flow diagram showing the distribution of the different pancreatic fistula grades

An overview of patient characteristics is provided in *Table* 1 and *Fig*. 1. A total of 108 patients were included in the final analysis.

**Table 1:** Clinical characteristics of patients with microbiological data

	No. of patients* (*n* = 108)
**Age (years)** †	65·0 (31–85)
**Sex ratio (M** : **F)**	70 : 38
**BMI (kg/m** ^**2**^ **)** ‡	25·6 (23·4–28·1)
**Diabetes**	8 (7·4)
**Histopathology**	
Ductal adenocarcinoma	52 (48·1)
Distal bile duct cancer	21 (19·4)
Chronic pancreatitis	17 (15·7)
Benign condition	12 (11·1)
IPMN	4 (3·7)
Other	2 (1·9)
**Preoperative bile duct stenting**	16 (14·8)
**Operation**	
Pancreatic head resection	61 (56·5)
Pylorus‐preserving Whipple	53 (49·1)
Classical Whipple	3 (2·8)
DPPHR	5 (4·6)
Distal pancreatectomy	47 (43·5)
**Duration of surgery (h)** ‡	5·5 (4·4–6·8)
**Grade of pancreatic fistula**	
Biochemical leak	10 (9·3)
B	49 (45·4)
C	49 (45·4)
**Wound infection**	9 (8·3)
**Clavien–Dindo grade**	
I	3 (2·8)
II	9 (8·3)
III	53 (49·1)
IV	32 (29·6)
V	11 (10·2)
**Relaparotomy**	33 (30·6)
**Somatostatin administration**	29 (26·9)
**Bacterial spectrum in fistula**	
Sterile	19 (17·6)
Non‐intestinal bacteria (e.g. *Streptococcus* spp.)	16 (14·8)
Enterobacterales	29 (26·9)
MDRE	32 (29·6)
Other intestinal bacteria (e.g. *Haemophilus parainfluenzae, Enterococcus* spp.)	12 (11·1)

* With percentages in parentheses unless indicated otherwise;

† values are median (range) and

‡ median (i.q.r.).

IPMN, intraductal papillary mucinous neoplasm; DPPHR, duodenum‐preserving pancreatic head resection; MDRE, multidrug‐resistant Enterobacterales.

### Characteristics of pancreatic fistula fluid

Of the 108 patients from whom a PF fluid swab was taken for microbiology, 89 (82·4 per cent) had an infected PF and 19 (17·6 per cent) had a sterile fistula (*Table* 1). The rate of infected fistula was higher after PD (58 of 61 patients, 95 per cent) than after DP (31 of 47, 66 per cent) (*P* < 0·001). The infected fistulas were polymicrobial (contained more than 1 bacterial strain), especially after PD (29 of 58, 50 per cent) compared with DP (8 of 31, 26 per cent) (*P* = 0·023).

A large number of different bacterial species were detected in the drain fluid after both PD and DP (*Table* 2). Approximately 74 and 34 per cent of the microorganisms detected after PD and DP respectively were Enterobacterales, among which 64 per cent (29 of 45) and 19 per cent (3 of 16) respectively were MDRE (*P* < 0·001).

**Table 2:** Bacterial species and microorganisms in fistula fluid after pancreatoduodenectomy and distal pancreatectomy

	PD (*n* = 61)	DP (*n* = 47)
**Intestinal bacteria**		
Enterobacterales	45 (74)	16 (34)
MDRE	29 (48)	3 (6)
*Escherichia coli*	23 (38)	10 (21)
*Klebsiella* spp.	19 (31)	8 (17)
*Proteus* spp.	6 (10)	3 (6)
*Enterobacter* spp.	13 (21)	1 (2)
*Citrobacter* spp.	5 (8)	0 (0)
Other intestinal microorganisms		
*Haemophilus parainfluenzae*	2 (3)	0 (0)
*Enterococcus* spp.	40 (66)	9 (19)
*Candida* spp.	29 (48)	5 (11)
*Bacteroides* spp.	3 (5)	3 (6)
*Prevotella* spp.	3 (5)	0 (0)
**Non‐intestinal bacteria**		
*Streptococcus* spp.	10 (16)	9 (19)
*Staphylococcus* spp.	15 (25)	28 (60)
*Corynebacterium* spp.	0 (0)	3 (6)
Other	7 (11)	8 (17)

Values in parentheses are percentages. PD, pancreatoduodenectomy; DP, distal pancreatectomy; MDRE, multidrug‐resistant Enterobacterales.

### Postoperative complications

A higher rate of Clavien–Dindo grade IV–V complications was associated with the detection of both Enterobacterales and MDRE in PF fluid (*Tables* 3 and 4). In subgroup analysis, this association was significant in patients undergoing DP, but not in those having PD (*Table* 3).

**Table 3:** Complications according to bacterial spectrum and type of operation

	Clavien–Dindo grade		
	I–III	IV–V	*P* *
**All patients**			0·004
No Enterobacterales	35	12	
Enterobacterales	15	14	
MDRE	12	20	
**DP**			0·014
No Enterobacterales	24	7	
Enterobacterales	5	8	
MDRE	1	2	
**PD**			0·094
No Enterobacterales	11	5	
Enterobacterales	10	6	
MDRE	11	18	

Values in parentheses are percentages. MDRE, multidrug‐resistant Enterobacterales; DP, distal pancreatectomy; PD, pancreatoduodenectomy.

* Fisher's exact test.

**Table 4:** Univariable logistic regression analysis of complications: Clavien–Dindo grade I–III *versus* grade IV–V

	Odds ratio	*P*
**Bacterial spectrum**		
No Enterobacterales	1·00 (reference)	
Enterobacterales	2·72 (1·02, 7·25)	0·045
MDRE	4·86 (1·87–12·85)	0·001
Parenchymal stiffness (soft *versus* hard) (*n* = 23)*	1·71 (0·33–9·93)	0·550
**Duct diameter (*n* = 76)**	0·96 (0·67–1·41)	0·829
**BMI**	0·97 (0·84–1·07)	0·974
**Age**	1·03 (0·93–1·06)	0·092
**Duration of surgery**	1·08 (0·82–1·34)	0·112
**PD *versus* DP**	1·60 (0·73–3·41)	0·203

Values in parentheses are 95 per cent confidence intervals. MDRE, multidrug‐resistant Enterobacterales; PD, pancreatoduodenectomy; DP, distal pancreatectomy.

In addition, a higher rate of clinically worse PF (grade C) was associated with both Enterobacterales and MDRE detected in PF fluid compared with non‐Enterobacterales/sterile PF fluid (*Tables* 5 and 6). In multivariable logistic regression analysis, only the presence of Enterobacterales and MDRE were independent risk factors for severe complications (OR 2·83, 95 per cent c.i. 1·04 to 7·87, *P* = 0·042; and OR 5·53, 1·74 to 16·86, *P* = 0·003, respectively) and grade C PF (OR 2·96, 1·05 to 8·15, *P* = 0·044; and OR 3·46, 1·16 to 10·62, *P* = 0·033, respectively).

**Table 5:** Development of pancreatic fistula according to bacterial spectrum

	Pancreatic fistula			
	Biochemical leak	Grade B	Grade C	*P* *
**Bacterial spectrum**				0·021
No Enterobacterales	6	28	13	
Enterobacterales	2	11	16	
MDRE	2	10	20	

MDRE, multidrug‐resistant Enterobacterales.

* Fisher's exact test.

**Table 6:** Univariable logistic regression analysis of pancreatic fistula: grade B *versus* grade C

	Odds ratio	*P*
**Bacterial spectrum**		
No Enterobacterales	1·00 (reference)	
Enterobacterales	3·13 (1·14, 8·65)	0·025
MDRE	4·32 (1·58, 11·76)	0·004
**Parenchymal stiffness (soft *versus* hard) (*n* = 23)**	0·71 (0·12–4·13)	0·714
**Duct diameter (*n* = 76)**	1·03 (0·76–1·45)	0·856
**BMI**	1·00 (0·82–1·13)	0·698
**Age**	1·03 (0·99, 1·07)	0·055
**Duration of surgery**	1·19 (0·96, 1·48)	0·110
**PD *versus* DP**	2·36 (1·03, 5·39)	0·045

Values in parentheses are 95 per cent confidence intervals. MDRE, multidrug‐resistant Enterobacterales; PD, pancreatoduodenectomy; DP, distal pancreatectomy.

The duration of drainage was not associated with the severity of PF (*P* = 0·902), or with the presence of intestinal microorganisms (*P* = 0·441) or multiresistant bacteria (*P* = 0·565). In the multivariable logistic regression analyses, the emergence of Enterobacterales, rather than other bacteria, was associated with patient age (OR 1·04 (95 per cent c.i. 1·00 to 1·08) per year; *P* = 0·029) and type of surgery (OR 5·32 (2·28 to 12·53) for PD (compared with DP); *P* < 0·001).

## Discussion

This study has demonstrated that PF fluid has a distinct intestinal bacteriological spectrum after both PD and DP. The pathogenetic mechanism behind the colonization of pancreatic collections and PF with intestinal bacteria after DP is currently unknown. However, understanding this mechanism may have important implications for effective treatment of PF, especially as the present study showed an association between distinct intestinal bacteria (Enterobacterales) in the PF fluid and more severe complications after DP.

Peripancreatic collections due to leakage from the pancreatic resection plane are known to be infected frequently. After pancreatic head resection and subsequent pancreatojejunostomy, such infection is typically attributed to the displacement of intestinal bacteria from the leaking anastomosis. However, why and how PFs and peripancreatic collections become infected after DP is unknown. At least in theory, haematoma at the resection plane may be a hotbed for bacterial growth. On the other hand, keeping the intra‐abdominal drain *in situ* for longer periods may result in ascending intra‐abdominal infection. However, in the absence of intraoperative damage to the intestine, neither theory can explain the occurrence of intestinal bacteria in PF fluid.

In the authors' view, one possible explanation for the presence of intestinal bacteria in PF‐associated collections is the natural bacteriological flora of pancreatic juice. Information in the literature regarding the bacterial content or flora of normal pancreatic juice in the healthy pancreas is sparse. For a long time, researchers believed that, owing to the presence of aggressive proteases and digestive enzymes, pancreatic juice would actually have antibacterial activity^8^. This activity was demonstrated against *E. coli, Shigella* spp., *Salmonella* spp. and *Klebsiella* spp., but not against *Bacteroides fragilis* or *Streptococcus faecalis*, for example^8^. The present authors acknowledge that an ideal study would obtain bacteriology swabs prospectively from PF fluid, regardless of suspected clinical infection, at early and late time points. However, such a study would necessitate leaving the drain *in situ* for longer in a control group of patients with no sign of infection in the PF fluid. This potentially unnecessary drainage may similarly lead to secondary, unwanted infection/contamination of the fistula fluid.

Endoscopic therapies have been used increasingly to drain parapancreatic collections, and gastroenterologists are therefore interested in the microbiological spectrum of pancreatic fluid collections. In this regard, Mönkemüller and colleagues^9^ showed that bacteria are readily detectable in patients with acute or chronic pancreatitis‐associated fluid collections. Furthermore, in a study of 26 patients who had surgery for chronic pancreatitis, Parida and co‐workers^10^ found bacteria in the pancreatic duct fluid of 11 patients. Bacteria were, without exception, present in the pancreatic fluid cultures of patients who had preoperative ERCP, as a result of ascending contamination from the intestine^10^. The most common organism observed was *E. coli*, followed by *Klebsiella pneumoniae*, and bacteria isolated from the wound were similar to those in the pancreatic fluid^10^. Although the patient cohort was small, this study^8^ clearly showed that pancreatic juice is frequently infected in patients with chronic pancreatitis who had a previous endoscopic intervention.

In a study of patients with pancreatic cystic lesions that were drained endoscopically, Li *et al*.^11^ also found intestinal bacterial strains, such as *Bacteroides* spp., *E. coli* and *Shigella* spp., to be present in cyst fluid. In the present study, it was interesting that in patients with DP and no preoperative manipulation of the ampulla or main pancreatic duct, and no intraoperative opening of any intestinal lumen, half of all organisms present in infected PF fluid were intestinal bacteria.

Another explanation for the presence of intestinal bacteria in PF fluid after DP may be bacterial translocation, an event known to occur during acute or chronic pancreatitis^12^, ^13^. Infection of peripancreatic collections during acute pancreatitis is ascribed to such a translocation process from the surrounding intestinal loops, such as colon. It is possible that such translocation occurs due to surgery‐induced, self‐limiting, acute pancreatitis, which may result from DP. Indeed, in the authors' clinical experience, short‐lived increases in serum amylase or lipase levels are common in the first 24 h after pancreatic resection, including resection of the tail. Such a translocation may be facilitated by disturbances in the microbiome of patients with pancreatic disease^14^, or by the presence of exocrine pancreatic insufficiency^15^.

What is the clinical consequence of the abundant presence of intestinal bacteria in PF fluid after DP? Undoubtedly, clinicians administer antibiotics to such patients with signs of sepsis or infection based on the antibiotic resistance profile of identified bacteria. An interesting question is whether bacterial translocation, which possibly occurs very early in the postoperative phase, may contribute to the generation of PF. Elucidating the potential impact of bacterial translocation on the healing process of the pancreatic resection plane may have key implications for preventing PF. Based on the present results, it is important to consider more aggressive therapeutic measures for patients with intestinal and/or multiresistant bacteria in the fistula fluid. Such measures could be more effective drainage of the peripancreatic collection and/or administration of antibiotics. However, antimicrobial therapy should be used only in the context of clinically suspected infection to avoid the emergence of multiresistant bacteria. Whether such an augmented intervention strategy would result in an improved clinical course needs to be analysed in future prospective studies.

This study has demonstrated that PF fluid after both PD and DP is frequently infected with intestinal bacteria. Understanding the pathogenetic mechanism behind the presence of intestinal bacteria in the fistula fluid after DP may help to improve the rate of infectious postoperative complications and contribute to knowledge on the development of PF.

## Supporting information


**Table S1** Risk factors influencing the occurrence of clinically relevant (grade B–C) pancreatic fistula and intestinal bacteria in the pancreatic fistula fluid

## References

[1] Tieftrunk E , Demir IE , Schorn S , Sargut M , Scheufele F , Calavrezos L *et al* Pancreatic stump closure techniques and pancreatic fistula formation after distal pancreatectomy: meta‐analysis and single‐center experience. PLoS One 2018; 13: e0197553. 10.1371/journal.pone.0197553 PMC5999073 29897920

[2] Denbo JW , Orr WS , Zarzaur BL , Behrman SW . Toward defining grade C pancreatic fistula following pancreaticoduodenectomy: incidence, risk factors, management and outcome. HPB (Oxford) 2012; 14: 589–593. 22882195 10.1111/j.1477-2574.2012.00486.x PMC3461384

[3] Bassi C , Marchegiani G , Dervenis C , Sarr M , Abu Hilal M , Adham M *et al*; International Study Group on Pancreatic Surgery (ISGPS). The 2016 update of the International Study Group (ISGPS) definition and grading of postoperative pancreatic fistula: 11 years after. Surgery 2017; 161: 584–591. 28040257 10.1016/j.surg.2016.11.014

[4] Frankard J , Rodriguez‐Villalobos H , Struelens MJ , Jacobs F . *Haemophilus parainfluenzae*: an underdiagnosed pathogen of biliary tract infections? Eur J Clin Microbiol Infect Dis 2004; 23: 46–48. 14669072 10.1007/s10096-003-1050-z

[5] Whiley RA , Beighton D , Winstanley TG , Fraser HY , Hardie JM . *Streptococcus intermedius*, *Streptococcus constellatus*, and *Streptococcus anginosus* (the *Streptococcus milleri* group): association with different body sites and clinical infections. J Clin Microbiol 1992; 30: 243–244. 1734062 10.1128/jcm.30.1.243-244.1992 PMC265033

[6] Dindo D , Demartines N , Clavien PA . Classification of surgical complications: a new proposal with evaluation in a cohort of 6336 patients and results of a survey. Ann Surg 2004; 240: 205–213. 15273542 10.1097/01.sla.0000133083.54934.ae PMC1360123

[7] Magiorakos AP , Srinivasan A , Carey RB , Carmeli Y , Falagas ME , Giske CG *et al* Multidrug‐resistant, extensively drug‐resistant and pandrug‐resistant bacteria: an international expert proposal for interim standard definitions for acquired resistance. Clin Microbiol Infect 2012; 18: 268–281. 21793988 10.1111/j.1469-0691.2011.03570.x

[8] Rubinstein E , Mark Z , Haspel J , Ben‐Ari G , Dreznik Z , Mirelman D *et al* Antibacterial activity of the pancreatic fluid. Gastroenterology 1985; 88: 927–932. 3882511 10.1016/s0016-5085(85)80009-3

[9] Mönkemüller KE , Harewood GC , Curioso WH , Fry LC , Wilcox CM , Morgan DE *et al* Biochemical analysis of pancreatic fluid collections predicts bacterial infection. J Gastroenterol Hepatol 2005; 20: 1667–1673. 16246183 10.1111/j.1440-1746.2005.04067.x

[10] Parida SK , Pottakkat B , Raja K , Vijayahari R , Lakshmi CP . Bacteriological profile of pancreatic juice in patients with chronic pancreatitis. JOP 2014; 15: 475–477. 25262715 10.6092/1590-8577/2256

[11] Li S , Fuhler GM , Bn N , Jose T , Bruno MJ , Peppelenbosch MP *et al* Pancreatic cyst fluid harbors a unique microbiome. Microbiome 2017; 5: 147. 29122007 10.1186/s40168-017-0363-6 PMC5680603

[12] Chen J , Huang C , Wang J , Zhou H , Lu Y , Lou L *et al* Dysbiosis of intestinal microbiota and decrease in paneth cell antimicrobial peptide level during acute necrotizing pancreatitis in rats. PLoS One 2017; 12: e0176583. 10.1371/journal.pone.0176583 PMC5404871 28441432

[13] Ammori BJ , Leeder PC , King RF , Barclay GR , Martin IG , Larvin M *et al* Early increase in intestinal permeability in patients with severe acute pancreatitis: correlation with endotoxemia, organ failure, and mortality. J Gastrointest Surg 1999; 3: 252–262. 10481118 10.1016/s1091-255x(99)80067-5

[14] Rogers MB , Aveson V , Firek B , Yeh A , Brooks B , Brower‐Sinning R *et al* Disturbances of the perioperative microbiome across multiple body sites in patients undergoing pancreaticoduodenectomy. Pancreas 2017; 46: 260–267. 27846140 10.1097/MPA.0000000000000726 PMC5235958

[15] Williams DA , Batt RM , McLean L . Bacterial overgrowth in the duodenum of dogs with exocrine pancreatic insufficiency. J Am Vet Med Assoc 1987; 191: 201–206. 3610795

