# Supplementary material for: Association of bacteria in pancreatic fistula fluid with complications after pancreatic surgery
Source: BJS Open. 2020 Apr 16;4(3):432–7. doi: 10.1002/bjs5.50272 (PMC7260411; doi:10.1002/bjs5.50272)
Supplement: Supplementary file 1 — Table S1 Risk factors influencing the occurrence of clinically relevant (grade B–C) pancreatic fistula and intestinal bacteria in the pancreatic fistula fluid [file BJS5-4-432-s001.docx]

**BJS5_50272**

**Association of bacteria in pancreatic fistula fluid with complications after pancreatic surgery**

**E. Demir, K. Abdelhai, I. E. Demir, C. Jäger, F. Scheufele, S. Schorn, K. Rothe, H. Friess and G. O. Ceyhan**

**Table S1** Risk factors influencing the occurrence of clinically relevant (grade B–C) pancreatic fistula and intestinal bacteria in the pancreatic fistula fluid

|  | | **Pancreatic fistula grade** | | | **Total** |
| --- | --- | --- | --- | --- | --- |
|  |  | **Biochemical leak** | **B** | **C** |  |
| Parenchymal stiffness | Very soft | 0 | 6 | 7 | 13 |
|  | Normal | 1 | 2 | 2 | 5 |
|  | stiff | 0 | 1 | 4 | 5 |
| Total | | 1 | 9 | 13 | 23 |
| Duct diameter (median) | | 1.65 | 1.5 | 1.6 |  |
